# Role of phytomelatonin responsive to metal stresses: An omics perspective and future scenario

**DOI:** 10.3389/fpls.2022.936747

**Published:** 2022-09-06

**Authors:** Skhawat Ali, Rafaqat Ali Gill, Muhammad Sohaib Shafique, Sunny Ahmar, Muhammad Kamran, Na Zhang, Muhammad Riaz, Muhammad Nawaz, Rouyi Fang, Basharat Ali, Weijun Zhou

**Affiliations:** ^1^Institute of Crop Science and Zhejiang Key Laboratory of Crop Germplasm, Zhejiang University, Hangzhou, China; ^2^Key Laboratory of Biology and Genetic Improvement of Oil Crops, The Ministry of Agriculture and Rural Affairs, Oil Crops Research Institute of Chinese Academy of Agricultural Sciences, Wuhan, China; ^3^Institute of Crop Sciences, Chinese Academy of Agriculture Sciences, Beijing, China; ^4^Faculty of Natural Sciences, Institute of Biology, Biotechnology and Environmental Protection, University of Silesia, Katowice, Poland; ^5^School of Agriculture, Food and Wine, The University of Adelaide, Urrbrae, SA, Australia; ^6^College of Natural Resources and Environment, South China Agricultural University, Guangzhou, Guangdong, China; ^7^Department of Agricultural Engineering, Khwaja Fareed University of Engineering and Information Technology, Rahim Yar Khan, Pakistan

**Keywords:** signaling molecule, sRNAs analysis, transcriptomics, genetic modification, ionomics

## Abstract

A pervasive melatonin (*N-acetyl-5-methoxytryptamine*) reveals a crucial role in stress tolerance and plant development. Melatonin (MT) is a unique molecule with multiple phenotypic expressions and numerous actions within the plants. It has been extensively studied in crop plants under different abiotic stresses such as drought, salinity, heat, cold, and heavy metals. Mainly, MT role is appraised as an antioxidant molecule that deals with oxidative stress by scavenging reactive oxygen species (ROS) and modulating stress related genes. It improves the contents of different antioxidant enzyme activities and thus, regulates the redox hemostasis in crop plants. In this comprehensive review, regulatory effects of melatonin in plants as melatonin biosynthesis, signaling pathway, modulation of stress related genes and physiological role of melatonin under different heavy metal stress have been reviewed in detail. Further, this review has discussed how MT regulates different genes/enzymes to mediate defense responses and overviewed the context of transcriptomics and phenomics followed by the metabolomics pathways in crop plants.

## Introduction

Heavy metals (HMs) stress has emerged as a major problem in a variety of terrestrial habitats around the globe. Due to heavy metals, widespread industrialization negatively influences the crop and soil productivity (Shahid et al., 2015). Metals presence in the soil disturbed the texture of soil, pH, reduces plant growth directly and/or indirectly by interfering with various molecular and physiological activities in crop plants (Hassan et al., 2017; Wang et al., 2019). Metals like Fe, Mn, Cu, Ni, Co, Cd, Zn, Hg, and As have been accumulating in soils for a long time as a result of anthropogenic activities like the waste of different industries, application of fertilizer, sewage disposal and smelting (Zhang and Wang, 2020). Metals are leached into groundwater or accumulated on the soil surface because of these activities (Dağhan and Ozturk, 2015; Hakeem et al., 2015; Basheer, 2018). Toxic metals cannot be removed from the atmosphere by natural processes; so, these metals are non-biodegradable. Some are static, unable to leave the area where they have gathered, while others are mobile and easily absorbed by plant roots (Ali et al., 2015b, 2018; Dehghani et al., 2016; Burakova et al., 2018).

Bioactive metals are classified into two classes based on their physicochemical properties: redox metals such as Mn, Cu, Fe, and Cr, and non-redox metals such as Zn, Cd, Al, Ni, and Hg (Jozefczak et al., 2012). By undergoing Haber-Weiss and Fenton reactions, redox metals can directly cause oxidative injury in plants, breakage of DNA strands, destruction of cell homeostasis, defragmentation of protein, photosynthetic pigment damage and, damage of cell membrane which can lead to death of cell (Flora, 2009). Non-redox active metals, on the other hand, trigger oxidative stress indirectly through a variety of mechanisms, including glutathione depletion, protein sulfhydryl group binding (Jozefczak et al., 2012), antioxidative enzymes inhibition, and induction of ROS-producing enzymes such as NADPH oxidases (Bielen et al., 2013). Plants use a variety of mechanisms to protect themselves from different sources of metals by developing tolerance. The cutting edge “omic tools” are an excellent model for understanding plants molecular mechanism of tolerance/susceptibility under the impact of various stresses, especially under HMs (Wang et al., 2020). Omic approaches used for HMs stress mainly include transcriptomics, proteomics, metabolomics and ionomics (Liu et al., 2020). Another mechanism is exclusive, meaning plants are only enabled when a certain level of metal toxicity is present. All responses fall into two categories: avoidance or tolerance (Krzesłowska, 2011; Shahid et al., 2015). Metabolomics, proteomics, and transcriptomics are common omics approaches used to interpret regulatory networks involved in responding to HMs tolerance in plants (Singh et al., 2016). The above-mentioned omics approaches, when combined with various functional genomic approaches, help to produce such verities which have the ability to tolerate against various abiotic stress conditions (Mosa et al., 2017). Plant growth regulators are chemical substances that control all aspects of plant production and growth (Ali et al., 2013a, 2013b). Several different plant growth-promoting rhizobacteria (PGPRs) have been used in the past to try to achieve efficient plant growth under abiotic stress (Ali et al., 2013a, 2013c).

Plant hormones (phytohormones) work as a messenger which can be either natural or synthetic chemicals, that control growth and development in response to environmental cues and are much effective even at very low absorptions (Rubio et al., 2009). Phytohormones work in various forms and activate various processes, including cell division (cytokinins) and cell development (auxins). Interestingly, plants have evolved several signaling pathways to overcome various stresses. One of them is mitogen-activated protein kinase (MAPK) synthesis (Arnao and Hernández-Ruiz, 2015, 2020). MAPK production regulates several critical stress-related hormones, genes, and chemicals that ultimately defend plants to survive against abiotic stresses (Arnao and Hernández-Ruiz, 2015). Plant hormones have been shown to promote the growth of non-plant microorganisms such as bacteria and fungi in many studies (Chatterjee et al., 2008; Levy et al., 2018; Kunkel and Johnson, 2021). They significantly affect on different physiological processes such as plant growth, development, and movement. Despite extensive research on the topic, no major breakthroughs have been made until now. The most well-known and essential phytohormones are melatonin, indole-3-acetic acid, gibberellic acid, kinetin, 1-triacontanol and abscisic acid.

Melatonin (MT, *N-acetyl-5-methoxytryptamine*) is a multi-regulatory chemical involved in seed germination, root growth, fruit ripening, senescence, yield, circadian rhythm, and stress response. Practically, it is found in all plant species (Hernández et al., 2015; Sun et al., 2015; Hardeland, 2016) and it is known as growth promoter and rooting agent (Chen et al., 2009a; Sarrou et al., 2014; Zhang et al., 2021). It plays a significant role in plant stress defense in addition to its role in plant growth. Plants can be exposed to stressful environmental conditions regularly. Plant species high in MT have been shown to have a higher stress tolerance ability. Exogenous therapy or ectopic overexpression of MT biosynthesis genes may also improve tolerance to a range of stressors, such as high temperatures, dehydration, salinity, radiation, and chemical challenges, all of which can produce the reactive oxygen species (Zhang et al., 2015). In this review, we have tried to compile all the knowledge about melatonin regulatory effects in plants and physiological functions of MT under different heavy metal stresses. Keeping in mind the importance of MT and its role in alleviating heavy metal stress in plants, recent knowledge about how MT regulates different genes/enzymes in response to abiotic stress, as well as the transcriptomics and phenomics background of MT in crop plants under stressful conditions, is presented in a trendy manner.

## Role of phytomelatonin in plants: An overview

Melatonin is a low molecular weight indole molecule found in all kingdoms of life, from prokaryotes to eukaryotes, from animals to plants (Arnao and Hernández-Ruiz, 2014). It was first discovered as an essential animal hormone involved in antioxidant acts, reproduction, circadian cycles, and innate immunity, among other biological processes (Shi et al., 2015). Since, the discovery of MT in Japanese morning glory (Pharbitis nil) in 1993, much progress has been made in understanding the function of melatonin in plants (Sun et al., 2021). Different studies have indicated that melatonin has unique physiological properties in plants, such as growth promotion and rhizogenesis induction, functioning similarly to *indolyl-3-acetic acid* (IAA) and auxin (Arnao and Hernández-Ruiz, 2014). Melatonin protects plants from abiotic stimuli such as HMs, UV radiation, salt, drought, and ambient temperature and contributes to the aging of leaves (Reiter et al., 2015). Melatonin is thought to have a primary role in plants as it operates as the first line of defense against internal and external oxidative stress by scavenging the ROS (Park et al., 2013; Arnao and Hernández-Ruiz, 2015; Altaf et al., 2021a). The nitrogen in the phytomelatonin molecule’s carbonyl group triggers the development of a new five-membered ring after it interacts with ROS (Kaur et al., 2015). Further, it enhances plant resilience to environmental challenges such drought, salt, cold, and oxidative stress, as well as delaying leaf senescence, whether exogenously administered or endogenously created (Arnao and Hernández-Ruiz, 2014; Zhang et al., 2014). It has also been considered as a broad-spectrum antioxidant and an endogenous free radical scavenger. It eliminates a variety of free radicals and ROS, such as nitric oxide (NO), peroxynitrite anion (NO_3_^−^), hydroxyl radical (OH^−^), and singlet oxygen (O_2_^−^). One of the most attractive features of this molecule, which sets it apart from most antioxidants, is its metabolites can also scavenge ROS and nitrogen species (Kwon et al., 2021).

**Figure 1:**
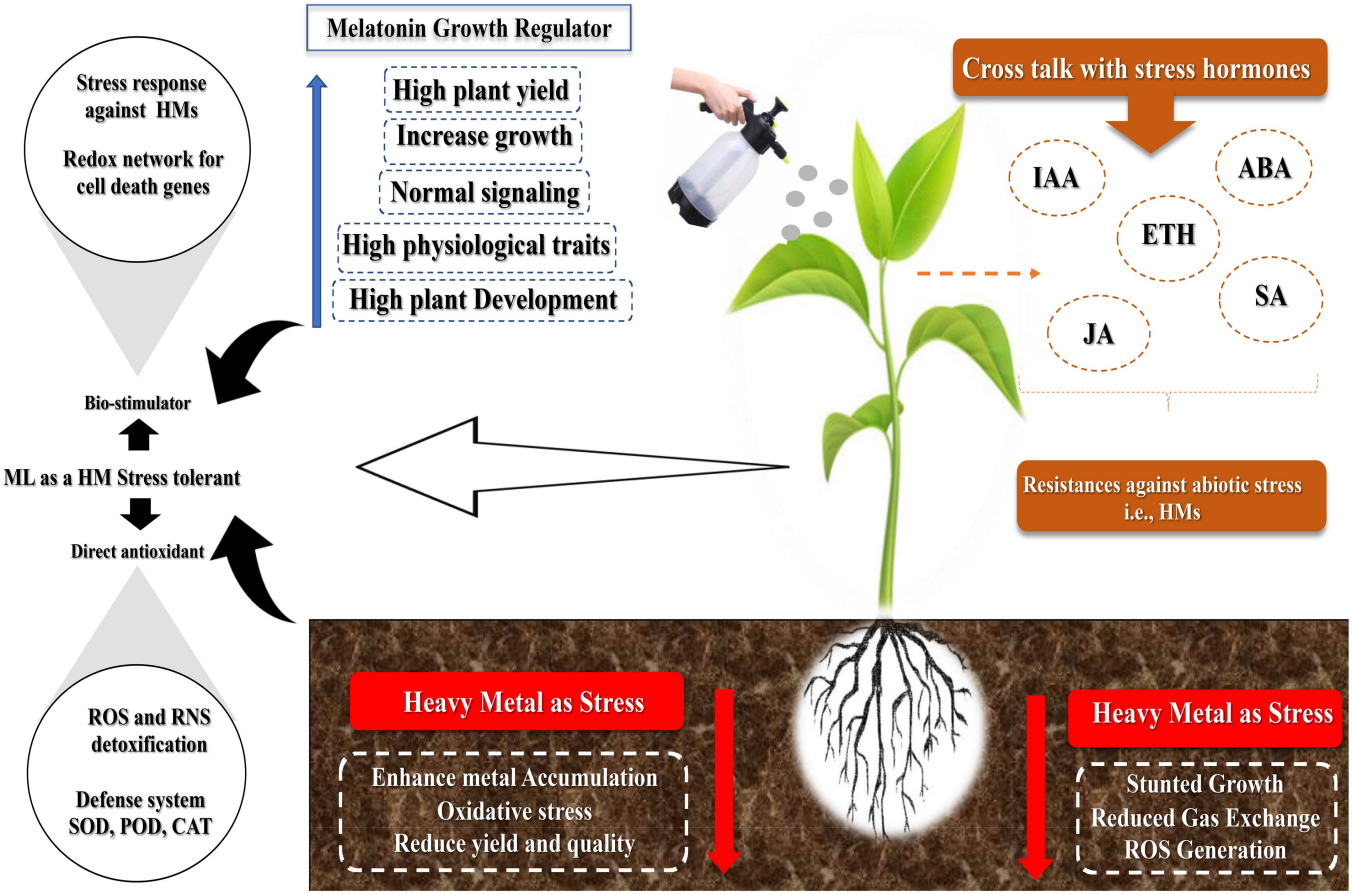
Graphical chart depicts the role of melatonin under abiotic stress conditions.

Exogenously applied MT has various effects, from substantial improvement to ineffectiveness or toxicity. The disagreement among researchers is due to the concentration used. At low and high doses, MT can have diverse roles in controlling plant growth and development in the same species. A low dose of MT (0.1 mM) promotes the root growth in wild leaf mustard (*Brassica juncea*), whereas a high dose (100 mM) inhibits its growth (Chen et al., 2009b). At low quantities, it promotes roots in cherry tissue culture, but at higher doses, it inhibits the root development (Sarropoulou et al., 2012). The high concentrations employed, specifically, 100 mM, could never be achieved in any plant because plant MT levels range from picograms to micrograms per gram of tissue. These values are several orders of magnitude higher than those in the human body. The toxic effects can occur when concentrations are too high. Melatonin levels were found to have a varied effect at low and high levels. However, high MT did not control any of the genes regulated by low MT (Weeda et al., 2014). This implies that MT can have various effects at low and high concentrations. At high concentrations, melatonin can drastically suppress ROS in cells, affecting ROS-dependent signaling and inhibiting cell development (Afreen et al., 2006). Figure 1 shows the graphical presentation of how MT alleviates abiotic stress in plants.

## Phenomics

Since, MT is soluble in both water and lipids and rapidly flows throughout the body to any watery area, it is largely employed as an antioxidant (Tan et al., 2013). However, many scientists have suggested that MT promotes plant growth in general (Arnao and Hernandez, 2019a). It has been found to increase the coleoptile length in canary grass, barley, and wheat (Hernández-Ruiz et al., 2005). Previously, maize seeds treated with MT showed greater seed vigor and quality and increased seed storage proteins (Kołodziejczyk et al., 2016). Another study found that coating soybean seeds with MT substantially boosted leaf development, plant height, number of pod plants^−1^, and number of seeds pod^−1^ (Wei et al., 2014). Melatonin has been demonstrated to play a comparable role in the promotion of vegetative growth and the regeneration of lateral and adventitious roots in etiolated *Lupinus albus* (Hernandez-Ruiz et al., 2004; Arnao and Hernández-Ruiz, 2007). While, treatment with MT resulted in increased seedling development, enhanced nutrient uptake efficiency, and improved nitrogen metabolism in cucumber plants, particularly under salt stress conditions (Zhang et al., 2017). According to recent investigations, MT administration also boosted photosynthetic activity, redox homeostasis, root growth and development, and seminal root elongation in barley, wheat, sweet cherry, and rice (Li et al., 2016; Liang et al., 2017; Zuo et al., 2017). Another potential element of MT application is its positive influence on photosynthesis and other growth-related parameters among different crops under various abiotic stress conditions (Meng et al., 2014; Wang et al., 2016a). Auxin, ethylene, cytokinin, gibberellins, IAA (indole 3-acetic acid), and brassinosteroids are all plant hormones that play an important role in maintaining growth and development of crop plants (Denancé et al., 2015). However, exogenous MT application can be used to modulate the effects of these plant hormones (Arnao and Hernández-Ruiz, 2017). Under salt conditions, exogenous MT dramatically increased the levels of endogenous abscisic acid (ABA) and gibberellic acid (GA) in cucumber seedlings, resulting in better salinity resistance (Zhang et al., 2014). The amount of cytokinin is steadily degraded in plants subjected to heat stress. However, once the plants treated with exogenous MT, an increase in the level of cytokinin biosynthesis can be detected, and therefore the increased cytokinin level in the melatonin-treated plants results in enhanced resilience to heat stress (Zhang et al., 2017). In a nutshell, the mechanistic principal of melatonin is to boost the antioxidant defense system; while, also increasing photosynthetic activity. Furthermore, several experts have claimed that MT has a major impact on total plant growth in the face of abiotic challenges with the least amount of impact on the environment (Tiryaki and Keles, 2012; Fan et al., 2015; Zhang et al., 2017; Ali et al., 2021).

Indole acetic acid is comparable in structure and function to the others (Hernández-Ruiz et al., 2005; Pelagio-Flores et al., 2012). In line with this, exogenous MT therapy boosts IAA synthesis (Wang et al., 2016b). On the other hand, both MT and IAA have been shown to enhance root development in a combined and similar manner (Yang et al., 2021). Exogenously administered MT increased IAA levels in *Brassica juncea* plants, resulting in improved root activity (Chen et al., 2009a). In *Mimosa pudica*, it showed a favorable effect on root organogenesis (Sun et al., 2021). Hence, MT is thought to play a significant role in plant’s physiological and biological processes. To summarize, MT can be considered a biological plant growth regulator that boosts a plant’s production capability. The concise plant growth alterations by MT in response to HMs stress are given in Table 1.

**Table 1:** Ameliorative effects of MT supplementation on growth, physiological, and biochemical attributes of plants grown under heavy metals toxicity.

Heavy metals	HMs dose	Plant species	Melatonin doses	Experiment type	Protective effect	References
Cadmium	10 mg/l	*Cyphomandra betacea*	0, 50, 100, 150, and 200 μmol/l	Nutrient solution cultivation	Low levels of MT (50 μmol/l) promoted the growth, while others behaved oppositely.	Lin et al. (2018)
Cadmium	200 mM	*Triticum aestivum*	50 mM	Petri dish experiment	MT caused an increment in reduced glutathione content and the oxidized glutathione ratio.	Ni et al. (2018)
Copper	80 μmol/l	*Cucumis sativus*	10 μmol/l	Hydroponic culture	MT improved Cu sequestration, carbon metabolism and ROS scavenging ability.	Cao et al. (2019)
Chromium	50 μM	*Brassica napus*	10 μM	Sand culture	Promoted ROS scavenging and chlorophyll stability, and modulated PSII stability.	Ayyaz et al. (2021)
Lead	800 mg/l	*Exophilic pisciphila* (isolated from the roots of *Arundinella bengalensis*)	50, 100, and 200 μM	Fungus growth medium	Significant reduction in malondialdehyde and oxygen free radicals; while, enhanced the activity of SOD.	Yu et al. (2021a)
Vanadium	40 mg/l	*Solanum lycopersicum*	100 μM	Hydroponic conditions	Restricted the production of ROS, improved photosynthesis, yield production, redox balance, mineral nutrients uptake and regulation of enzymes.	Altaf et al. (2021b)
Boron	50, 200 μM	*Triticum aestivum*	100 μM	Modified nutrient media	Scavenged ROS, improved contents of N, P, total soluble carbohydrates, enzymatic and non-enzymatic antioxidants.	Al-Huqail et al. (2020)
Nickel	50 μM	*Solanum lycopersicum*	100 μM	Hydroponics culture	Reduced Ni-induced growth damage and ROS production, boosted root architecture, nutrient uptake, and gas exchange attributes, and reduced Ni-accumulation.	Altaf et al. (2021a)
Iron	low-Fe (1/10^th^ of normal supply) and High-Fe (3-times of normal supply)	*Cucumis sativus*	100 μM	Hydroponics	MT played dual role in iron uptake by increasing the levels of *FRO2* and *IRT1* under low and high Fe stress, respectively.	Ahammed et al. (2020)
Selenium	50, 100 and 200 μM	*Brassica napus*	50 and 100 μM	Nutrient solution	MT improved biomass gain, pigment contents, PSII photochemical efficiency (Fv/Fm), boosted enzymatic antioxidants, proline, and free amino acids.	Ulhassan et al. (2019)
Cadmium	35 μM	*Solanum lycopersicum*	100 μM	Nutrient solution	MT application effectively reduced the cadium-induced phytotoxicity by enhancing root activity, growth attributes, and root morphological features.	Altaf et al. (2021c)
Arsenic	25 μM	*Camellia sinensis*	100 μM	Nutrient solution	Melatonin reduced As accumulation, ameliorated oxidative stress, boosted biosynthesis of anthocyanin.	Li et al. (2021)
Lead	50 μM	*Carthamus tinctorius*	100, 150, 200 and 300 μM	Nutrient solution	MT reduced translocation roots and Pb uptake to above-ground parts of safflower seedling.	Chamanara et al. (2019)
Cadmium and Aluminum	Cd (25 μM), and Al (25 μM)	*Brassica napus*	50, 100 μM	Nutrient solution	MT protectEthe photosynthetic apparatus from Al and Cd induced harms and limits the transfer of Al and Cd.	Sami et al. (2020)
Cadmium	100 μM	*Solanum lycopersicum*	100 μM	Hydroponic conditions	MT encouraged the biosynthesis of downstream sulfur metabolites such as γ-EC, cysteine, -EC, 2-CP, GSH, 2-CP, and PCs under Cd stress.	Hasan et al. (2019)
Copper	100 μM	*Brassica napus*	100 μM	Nutrient solution	MT reduced the levels of CuSO_4_-induced proline content and oxidative stress.	Kholodova et al. (2018)
Copper	80 μM	*Cucumis satvus*	10 μM	Nutrient solution	MT reduced copper toxicity by enhancing carbon metabolism, copper sequestration, and ROS scavenging.	Cao et al. (2019)

## Ionomics

Ionomics is the study of accumulating metalloids, non-metals and metals in living organisms, regarding the accumulated minerals, could be essentials and nonessential (Ayub et al., 2021). Plant ionomics has applied for various kinds of research, such as physiological, evolutionary, and ecological. Furthermore, the elemental analogs measuring can improve data quality with ionomic approaches (Watanabe et al., 2007; Chen et al., 2009b; Campos et al., 2021).

### Macro and micro elements

The macro and micro elements present in the plants which efficaciously promoted plants growth and many others boosted plant’s activities at metabolic and physiological levels. Various biotic and abiotic stress conditions can disturb plant behaviors physiologically and genetically. Therefore, these macro and micro elements should be affected by these changes. Thus, ionomics study has provide a reliable presumption regarding the ion concentration changes and various other pathways which control the elements in plant’s body. This accomplished that ionomics can be categorized as a superior tool for distinguishing amendments in the plants physiology with interrelation of environment (Kanwar et al., 2018). Besides that, plant ionomics can be associated to the difference of phylogenetic relationships (Watanabe et al., 2007). The ionomics application (use of mutants) has focused on isolation and gene characterization which were responsible for homeostasis and transmission of different plant elements (Chao et al., 2011; Duan et al., 2017). However, the most important benefit of ionomics implementation in plants has to enhance acomprehensive perception of elemental dynamics.

To reduce the toxicity of HMs, ionome (mineral nutrients) plays a major role, including N, P, K, Ca, S and Mg; along with some trace metals such as Fe, Cu, Mn, Mo, Co, and Zn. Nitrogen (N) is the main essential nutrient, composed of hormones, nucleic acids, proteins, and vitamins. It can alleviate the toxicity of heavy metals by promoting the photosynthetic competency by increasing the chlorophyll synthesis, boosting N-comprised metabolites such as GSH, proline and by increasing of antioxidant enzymatic activities (Lin et al., 2011). The other essential nutrient is phosphorus (P), comprised of nucleic acids and cell membrane, and especially imperative for reaction of phosphorylation. Phosphorus (P) can alleviate metal toxicity by diluting and decreasing metal mobility through metal phosphate complex (Sarwar et al., 2010). Additionally, P can also contribute in the prevention of membrane damage and increase the level of GSH content, thus conferring plant tolerance against metal stress (Wang et al., 2009a). On the other hand, calcium (Ca) has been involved in regulating metabolic activities as well as the intracellular and channel-based Ca-binding sites of Cd^2+^ and Ca^2+^ (Lauer Júnior et al., 2008). Further, Ca can take the place of the Cd that is present in the outside medium. Hence, plant development has been influenced, although Ca has shown a reduction in heavy metal-induced deficiencies (Farzadfar et al., 2013). In another study, Khan et al. (2009) reported that AsA–GSH cycle enhanced by 40 mg. S. Kg^−1^, thereby in mustard plant Cd toxicity reduction was obtained by Anjum et al. (2008). The accumulator plant species were more competitive against the stressed caused by Na, Ni and As based on the different adaptation strategies for excessive stress of these elements, which might form an enlarge variations of their concentration between various kinds of species (Pollard et al., 2014; White et al., 2017). Researchers have revealed that endogenous/exogenous application of MT could contribute in various plant species under salinity environment to enhance K^+^/Na^+^ homeostasis (Li et al., 2012). Most of scholars have investigated the strong correlation among salt tolerance and cellular K^+^ retention of the different kinds of plants, namely sweet potato (Yu et al., 2016), wheat (Cuin et al., 2008), halophytes (Bose et al., 2015), Brassica (Chakraborty et al., 2016), and poplar (Sun et al., 2009b).

### Influence of MT on ionomics

The influence of MT on ionomics has been reported in a recent study (Yu et al., 2018a). It was determined that depletive salt effect on the contents of Mg^2+^, K^+^, and Ca^2+^ in the different tissues reversed as MT exogenous application (root/leaf: 0.5/100 mM). Interestingly, MT distinctly reduced the Na^+^ significantly in the leaf tissues of Xu-32 under the salinized conditions but in the stem and root tissues Na^+^ content was increased and, MT in the absence of NaCl stress did not show any modifications of elemental levels (Yu et al., 2018b). Additionally, in sweet potato, MT maintained the K^+^/Na^+^ homeostasis, contributed to PM HC–ATPase activity and enhanced the energy state.

The effect of water deficits was evaluated on enzymes activities and genes transcriptional abundance included in N metabolism (Meng et al., 2016; Huang et al., 2018a,b). However, MT effect on the regulation of transcriptional genes in terms of N metabolism, uptake, and reduction under the drought stress thoroughly has not been studied. Previously, Dijkstra et al. (2015) investigated the absorption and transformation of N by implementing the ^15^N tracers. Therefore, Liang et al. (2018) applied some tracers under drought conditions to estimate MT impact on the nutrients uptake in plants. The exogenous MT has a modulating effect on the elements of plants and stress mitigation with the help of regulating those elements. For example, MT improved the reductions in Zn, Mg, Fe, Cu, Mn, K, P, and S concentrations. Meanwhile, it further increased the concentration of Ca and B in maize seedlings at low temperature conditions (Turk and Erdal, 2015). Thus, MT could be the most important hormone which can be helpful for plants to improve the drought tolerance by uptake process of mineral elements. This review proposes that the positive effect of MT prefers more opportunities related to agriculture, plants can be developed with higher drought tolerance and promotes an adaptation capacity for future environmental problems.

## Transcriptomics

Plant functional genetics provides new horizons for studying the molecular mechanisms behind the production of critical regulatory molecules (Thao and Tran, 2016; Abdelrahman et al., 2018). Transcriptomic approaches involve the study of the “transcriptome” of an organism. The transcriptome is a set of entire RNA molecules expressed under a particular circumstance spatially and temporally (Milward et al., 2016). Understanding the plant functional genetics by post-translational studies is getting routine research after the high-throughput sequencing technologies became approachable and much cheaper than before. These studies have a great promise to provide critical information about the molecular mechanism of synthesis, regulation, and metabolic pathways of action of products, by-products, and various critical regulatory molecules in plants (Pang et al., 2021). Transcriptomic approaches are one of the main pillars of molecular genetics, by the wealth of which scientists can determine real-time changes happening after the translation of genetic information imprinted in the plant cells (McGowan and Fitzpatrick, 2020).

Transcriptomic studies cover all aspects of RNA transcripts, their synthesis, expression, and their regulation. These studies also include their trafficking, structures, splicing patterns (mediated by spliceosome), and modifications at the post-transcriptional level (Liang, 2013). There are several types of transcripts; out of them, messenger RNA (mRNA), small RNA (sRNA), long noncoding RNA (lncRNA), and micro-RNA (miRNA) are remarkable (Milward et al., 2016). With the advent of cheap sequencing technologies, we can use these high-throughput methods for the expression analysis of transcripts under the particular conditions (Wang et al., 2009b). This knowledge is prime for linking phenotype to genotype. Several studies are available that reports transcriptional changes in plants under the specific physiological or pathological circumstance.

Plants are consistently encountered by various biotic and abiotic stresses (Małkowski et al., 2019). Some of these stresses are natural, while some result from anthropogenic activities. Heavy metals (HMs), the prime source of toxicity in plants, are being prolonged accumulated in soils through various activities (Chai et al., 2019). These activities include improper disposal of industrial waste, extensive fertilizers, non-treated sewage disposal to agricultural soil (Chai et al., 2019; Lominchar et al., 2019). These activities result in immobile, non-biodegradable, and toxic levels of HMs on the soil surface and ground water (Ahmad et al., 2016). Plants can take heavy metals and lead to toxicity in them (Mustafa and Komatsu, 2016). Plants can uptake HMs by different biological processes by their roots, using either diffusion, endocytosis, or metal transporters for this task (Zhao et al., 2021). Plants are also appreciated as bio-accumulators of HMs (Chaffai and koyama, 2011; Nawaz et al., 2016). Nevertheless, some metals are essential for plants, but their accumulation, when exceeding a specific limit, causes toxicity (Zhao et al., 2019). If animals eat these plants, toxicity can also be carried to their bodies, which can be poisonous in several ways. Effects and symptoms of HMs toxicity in plants include chlorosis, reduced growth, root death (browning), and plant wilting (Varma, 2021).

Ever since discovering melatonin in plants, research related to its role in plant homeostasis and regulation of stress-related signals is going on in many laboratories of the world (Kanwar et al., 2018). Its exogenous application is also remedial against infection from various pathogens. There are direct and indirect references that MT involvement in disease resistance in plants. However, its synthesis and regulation at the transcriptomic level is rarely summarized (Hoang et al., 2017; Abou-Elwafa et al., 2019; Liu et al., 2019). Here, this article will present a comprehensive overview of transcriptomic approaches used to understand the molecular genetics of phytomelatonin production, its regulation and involvement in plant regulatory mechanisms.

The cutting edge “omic tools” are an excellent model for understanding plants molecular mechanism of tolerance/susceptibility under the impact of various stresses, especially under HMs (Wang et al., 2020). Omic approaches used for HMs stress mainly include transcriptomics, proteomics, metabolomics and ionomics (Liu et al., 2020). The transcriptome is an entire set of transcripts, transcripted primarily in the form of mRNA, form the genome of an organism under certain conditions. Proteomics is the study of expressed proteins, their modification after translation, their localization, mode of action and their docking, translated from the transcriptome of an organism under a defined environmental condition (Abou-Elwafa et al., 2019). Metabolomic studies include identification, characterization, and quantification of metabolites (produced by cellular regulation, under the effect of external stimuli) and all metabolomic activities, and due to translated proteins, in the effect of conditions provided to plants, likewise economics is the quantitative measurement of production, accumulation of ions in an organism under the effect of external stimuli (Mustafa and Komatsu, 2016). Several biochemical pathways are directly and indirectly activated/suppressed after signal transduction of HMs in plants (Mustafa and Komatsu, 2016; Yu et al., 2021b).

### RNA sequencing and HMs tolerance: A prospective of MT-related genes

Plants produce differential expression of a set of the transcriptome under the abiotic stress of HMs, which is of significant interest for determining the genes involved in HMs tolerance and susceptibility (Duhan, 2021). RNA sequencing (RNA-seq) is an essential investigation to elucidate the regulation of gene expression at the molecular level. RNA-seq provides data about differential expression of genes and exposes critical biological processes involved in tolerance mediated by plants for HMs (Shahid et al., 2014; Lee and Back, 2017a). Several studies reported that the large set of genes were involved directly or indirectly in tolerance of HMs. These genes are either directly involved in signal transduction to produce metabolites that result in tolerance or indirectly regulate the production of other transcripts (Lee and Back, 2017b; Duhan, 2021). In broad terms, HMs stress-induced genes (transcripts) can be classified based on their functionality and two distinct groups can be made; one includes the functional genes, and the other is regulatory genes. The functional group of the transcript as a single or whole (without inducing the effect of another gene) encodes important compounds which play a critical role in the tolerance against HMs (Nawaz et al., 2016; Wilson et al., 2019). The functional group includes transcripts that mainly encode sugars, alcohols, and several amines. The second class of transcripts includes the stress-related gene involved in the regulatory network of different transcription factors (TFs) which work in groups or individually and involved in the regulation that led to HMs stress tolerance (Table 2; Wilson et al., 2019). Hence it comprises of gene network and has much more importance in understanding the broader picture. These TFs are often found in clusters in the plant genome and belongs to the multi-gene family (Table 2). These genetic regulators have unique expressional control of a single to several genes by binding at a specific site in promotor, acting as a cis-acting binding element to induce transcription. Otherwise, the gene is not expressed anyway (Cheng et al., 2018). This DNA binding class has a unique protein domain that acts as a cis-regulatory element *via* protein-to-protein interaction, which ultimately results in oligomerization of these transcriptional factors with several other regulatory genes (Wilson et al., 2019). This transcriptional complex-conjugate system is often referred as “regulon.”

**Table 2:** TFs in response to heavy metal stresses (Li et al., 2022).

Family	Gene	Heavy metal	Function	References
WRKY	*AtWRKY12*	Cadmium	It represses *GSH1* expression to negatively regulates Cd tolerance in Arabidopsis	Han et al. (2019)
	*AtWRKY13*	Cadmium	It activates *PDR8* expression to positively regulate Cd tolerance in Arabidopsis.	Sheng et al. (2019)
	*AtWRKY13*	Cadmium	It activates DCD during cadmium stress.	Zhang et al. (2020)
	*AtWRKY47*	Aluminum	It confers aluminum tolerance *via* regulation of cell wall modifying genes.	Li et al. (2020)
	*AtWRKY6*	Arsenic	It restricts arsenate uptake and transposon activation in Arabidopsis.	Castrillo et al. (2013)
MYB	*OsMYB45*	Cadmium	It is highly expressed under Cd stress. Mutation of *OsMYB45* resulted in hypersensitivity to Cd treatment	Hu et al. (2017)
	*SbMYB15*	Cadmium, Nickel	It confers Cd and Ni tolerance in transgenic tobacco.	Sapara et al. (2019)
	*AtMYB4*	Cadmium	It regulates Cd-tolerance *via* the coordinated activity of improved anti-oxidant defense systems.	Agarwal et al. (2020)
	*AtMYB72*	Zinc and Iron	It is more sensitive to excess Zn or Fe deficiency than wild-type	Van de Mortel et al. (2010)
	*DwMYB2*	Iron	The translocation of iron from root to shoot is affected by the *DwMYB2*.	Chen et al. (2006)
bZIP	*GubZIP*	Cadmium	It is expressed specifically in different tissues under Cd stress	Han et al. (2021)
	*bZIP19,23*	Zinc	It controls the plant zinc status	Lilay et al. (2018, 2021)
	*RsbZIP010*	Lead	It downregulates the expression under Pb stress.	Fan et al. (2019)
HSF	*VuNAR1*	Aluminum	It regulates Al resistance by regulating cell wall pectin metabolism.	Lou et al. (2020)
	*ZAT6*	Cadmium	It activates PC–related gene expression and directly targets GSH1 to positively regulate Cd accumulation in Arabidopsis.	Chen et al. (2016)
	*PvERF15*	Cadmium	It forms a Cd-stress transcriptional pathway.	Lin et al. (2017)
	*HIPP22*	Cadmium	It binds to the promote the regions of the *HIPP22* and *HIPP44*.	Zhang et al. (2019)

**Figure 2:**
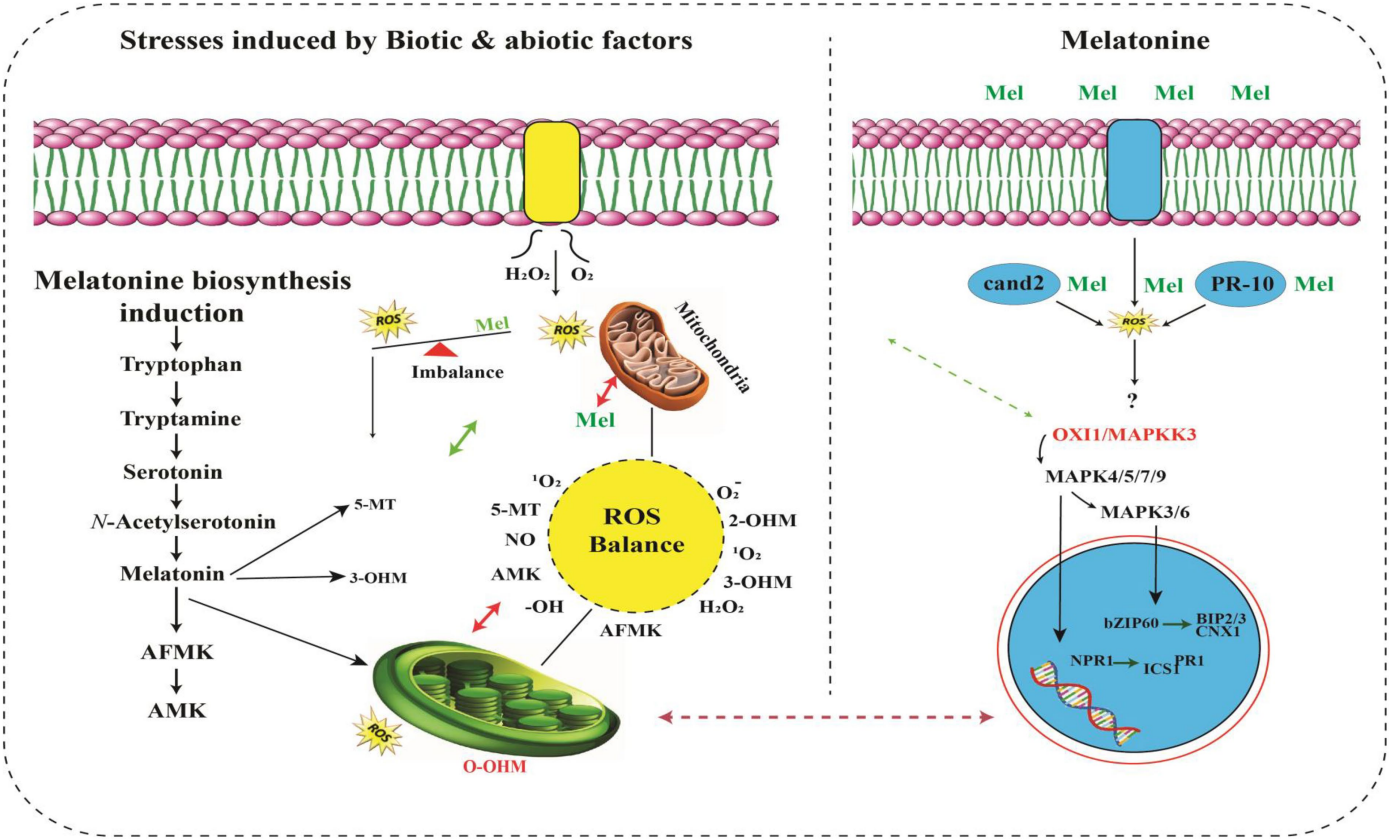
Overview of signaling pathways of melatonin and its metabolites (Back, 2021). MT activates MAPK cascade through *OXI1/MAPKKK3–MAPKK4/5/7/9–MAPK3/6*. MAPK activation induces translocation of the SA receptor *NPR1* into the nucleus to interact with several transcription factors, resulting in abiotic stress tolerance. Exogenously applied MT attenuates endoplasmic reticulum stress damage by increasing the expression of *BIP2, BIP3* and *CNX1* genes through the *bZIP60* transcription factor. Kinase (RLK) works as a MT receptor responsible for further activation of the *MAPK* cascade. ROS burst occurs from *RBOH* under stress conditions. Thus, ROS are powerful inducers of *de novo* melatonin biosynthesis. MT further metabolizes into *AFMK, AMK, 5-MT, 2-OHM, 3-OHM*, which are potent antioxidants. Melatonin and its metabolites efficiently scavenge a range of *ROS/RNS* to maintain cellular ROS balance. Unlike ROS-mediated *MAPK* activation upon stress (Jalmi and Sinha, 2015), melatonin-mediated *MAPK* activation is independent of ROS, indicating that melatonin functions downstream of the ROS burst (Lee and Back, 2017a). In this figure, solid arrows indicate confirmed functions; dashed arrows indicate steps not yet demonstrated.

A strong connection exists between raised MT levels and HMs stress tolerance (Zhang et al., 2022). MT greatly scavenges the reactive nitrogen species (RNS) along with reactive oxygen species (ROS; Gao et al., 2020), that produced excessively during routine photosynthesis process and respiration, as a prime functionality of plants. Plants critically utilize raised melatonin levels for scavenging elevated levels of RNS and ROS, hence providing tolerance to HMs stress otherwise which causes severe damage (Aslam et al., 2017; Lominchar et al., 2019; Zhang et al., 2020). The production of MT is also consistent with the upregulation of genes that produce stress-tolerant enzymes. These enzymes include *serotonin N-acetyltransferase* (SNAT), *tryptophan hydroxylase* (TPH), *N-acetylserotonin O-methyltransferase* (ASMT) and *tryptophan decarboxylase* (TDC), that are constantly produced in HMs stress tolerant plants. Fungal specie, i.e., *Exophiala pisciphila*, highly tolerant to HMs stress was isolated from smelting site of old mine in China. Yu et al. (2015) also reported that this specie’s transcriptomic analysis proved MTs involvement in the Salinity stress. ASMT, SANT and TDC are the enzymes regulating the biosynthesis of MT, were significantly upregulated under the stress of HMs like Cd (Chandramouli and Qian, 2009), indicating direct evidence of the connection of melatonin in HMs stress tolerance. It strongly suggests that melatonin has positive involvement with the enhanced HMs stress in plants. Figure 2 shows the MTs structure and signal transduction of HMs for its production using SNAT, TPH, ASMT, and TDC enzymes.

Several transcriptional families (TF) families are characterized in response to abiotic stresses in plants like HMs stress (Table 2). Here we provide a critical link between the production of MT and the contribution of these TFs families in response to HMs stress. Several TFs families are involved in MT production as a stress reliever in the plants under HMs stress (Liu et al., 2020). The transcriptional families that are greatly influenced by HMs stress are *AREB/ABF*, *MYB*, *AP2/EREBP*, *WRKY*, *bHLH*, *bZIP*, *MYC*, *HSF*, *DREB1/CBF*, *NAC*, *HB*, *ARID*, *EMF1*, *CCAAT-HAP2*, *CCAATDR1*, *CCAAT-HAP3*, *CCAAT-HAP5*, *C2H2*, *C3H*, *C2C2-Dof*, *C2C2-YABBY*, *C2C2-CO-like*, *C2C2-Gata*, *E2F-DP*, *ABI3VP1*, *ARF*, *AtSR*, *CPP*, *E2F-DP*, *SBP*, *MADS*, and *TUB* (Liu et al., 2020). These TFs are also famous for other abiotic stress inductive, and some of them also play a significant role in biotic stress tolerance. These TFs are regulon of various transcriptional activities in the broader picture after HMs stress induction in plants. In addition, these transcriptional activities lead to stress tolerance or stress resistance in plants. Table 2 represents the summary of TFs involved in the regulation of HMs stress in plants.

### Genomic approaches for HMs stress tolerance and MT synthesis

Comparative genomic approaches have provided critical inputs for crop improvement, especially by identifying biotic and abiotic stress related genes, quantitative trait loci (QTLs) and figuring out metabolic pathways (Frukh et al., 2020). Melatonin synthesis is directly related to heavy metals stress tolerance and critical for plant survival. Severe loss to crop yield was observed due to HMs stress and, more importantly, cause serious health issues when HMs accumulated plants were eaten up by animals or humans. Genomic approaches use various tools to identify key regulatory genes that are more or less associated with plant melatonin synthesis under the devastating stress of HMs, hence play an important role in plant physiology (Rajasundaram and Selbig, 2016). These approaches are also helpful in understanding the underlying molecular and physiological mechanisms of HMs tolerance. Genomics involve genome-scale studies of genetically diverse plants, using several genome wide transcriptomic approaches, gene expression analysis at the whole genome level, and discovery of novel genes related to various stresses like HMs.

Melatonin synthesis at genome level is controlled by a complex network of enzymes (Singh et al., 2016). Tryptophan, which is an essential amino acid, is responsible for melatonin production using six enzymes. These critical enzymes are *tryptophan decarboxylase* (TDC), TPH, *tryptamine 5-hydroxylase* (T5H), *serotonin N-acetyltransferase* (SNAT), *acetylserotonin-Omethyltransferase* (ASMT), and caffeic acid O-methyltransferase (Tripathi and Poluri, 2021). The genes responsible to produce these enzymes have been characterized and cloned for several plants. Very first SNAT gene was mapped in rice, which is also a model plant besides Arabidopsis. Several studies reported that SNAT was the penultimate enzyme and involved in final steps of melatonin production (Zhan et al., 2019). As many plants are whole genome sequenced, and researchers have benefited from the availability of high-quality genomic data to find homologs of these enzymes. SNAT homolog was also reported in other species, including alga laver, cyanobacteria, apple, Arabidopsis, grapevine (Tripathi et al., 2021). Furthermore, genome-wide studies have revealed that presence of these enzymes is diversified, with varied in frequency of existence in different plants.

Serotonin N-acetyltransferase is a regulatory enzyme for MT production as it maintains the production level of MT in response to stress (Reiter et al., 2015). Comparative genomic approaches revealed that different species have varied modular activities of SNAT, and it has distinct thermophilic properties. SNAT is primarily regarded as heat resistant enzyme having different temperatures for its catalytic activity in different plant species. Thus, it is anticipated that its part in heat stress tolerance, providing evidence of the role of melatonin in abiotic stress tolerance. Furthermore, ectopic overexpression of *MzSNAT5* in Arabidopsis reported increased melatonin concentration and improve drought tolerance. Moreover, when the SNAT production in rice was suppressed, the adverse effects were observed on plant growth, and low melatonin levels (Tripathi et al., 2021). It was certain that low melatonin levels have penalty of increased susceptibility to abiotic stresses and ultimately low yield levels. Melatonin, not only involved in abiotic stress tolerance but also correlated with biotic stresses. Bio-synthetic inhibition of G*hSANT1* and other melatonin related genes have compromised the resistance conferred by cotton against phytopathogenic bacteria. Thus, SNAT is an essential compound controlling melatonin synthesis in plants and has a significant role in abiotic stress and biotic stresses (Jayarajan and Sharma, 2021).

## Proteomics

Proteomics emerged as cutting-edge tool for understanding the synthesis, functionality, and expressional characterization of proteins at whole genome level (Yu et al., 2018a; Wang et al., 2018). Proteomic studies elucidate differential expression of proteins under various stresses like HMs and characterize them at cell, organ and tissue level as well as provide insights into network modulation of related proteins. Hence, emulating structural models identify HMs stress-tolerant material in other species (Pardo-hernández et al., 2021). Using proteomic approach is one of the prime studies for understanding the fate of a compound and its molecular mechanism of action, interaction at post translational level (Pardo-hernández et al., 2021). This involves whole protein level research related to our molecule of interest. Although genomic analyses significantly contributed to our understanding of basic gene functionality and how genes are translated to proteins, many puzzles for protein fate remained a challenge until we started to study whole proteins at genome level. This is because although the gene is transcribed and translated into protein, but the protein stability, folding, interaction with other proteins, and localization are critical for its functionality. Hence, in depth proteomic studies elucidate the target proteins (Rajasundaram and Selbig, 2016; Tripathi and Poluri, 2021) that directly or indirectly take part in melatonin production, provide interaction pathways, and provide better understanding on HMs stress tolerance.

Direct involvement of proteins in HMs stress tolerance/susceptibility is well known, as tolerance involves proteomic changes in plants. Therefore, with the use of proteomic approaches in plants, we exploited proteins involved in regulating HMs stress (Sage and Kubien, 2007). Furthermore, these studies helped a lot for deciphering the proteomic signals for perception of stress and initiation of signaling cascade that ultimately, with help of network changes at transcriptional and metabolomic level, provides tolerance against HMs-induced phytotoxicity (Mateos-Naranjo et al., 2008; Zhan et al., 2019). It has been categorized as indoleamine compound, synthesized as derivate of tryptophan (Yu et al., 2018a; Kanwar et al., 2018). Tryptophan is dominantly found in almost all higher plants. Besides providing tolerance against HM stress, melatonin also provides tolerance against various other abiotic stresses, like salinity, chilling, and osmatic stresses (Borges et al., 2019; Peharec Štefanić et al., 2019). Many researchers are working on for elucidating the molecular biochemistry of melatonin at proteomic level for its mitigation potential against HMs stress. Melatonin has been reported to improve antioxidant levels in diverse plants like wheat, tomato, and apple, hence reducing the ROS damage, providing tolerance to various stresses (Yu et al., 2018). This review offers insights how melatonin provides tolerance against HMs stress at proteomic level.

Reactive oxygen species (ROS)-scavenging proteins are critical for inducing tolerance against HMs stress. It was observed that under HMs stress, tolerant plants have an abundance of ROS scavenging proteins that performs series of chain reactions to MT homeostasis (Borges et al., 2019). Several metabolic pathways are significantly modulated under HMs stress like rate of respiration, metabolism of sulfur and nitrogen, and rate of photosynthesis. Under HMs stress, the plant power producing apparatus boosts the production of reducing agents like *FADH2*, *NADPH* and *NADH*, which lead to higher production of ATP (energy molecule of plants) to provide HMs stress tolerance. ROS scavenging is also correlated with higher production of reducing agents and increased melatonin production. For example, exogenous application of MT to HMs stressed plants have improved osmoregulation and increment in photosynthetic rate, antioxidants, and carotenoids compounds (Yu et al., 2018b). Heavy metals stress tolerance has evolved many key regulatory processes due to the elevation of MT in planta. These compounds like 1, 2 oxygen enhancer protein (OEP), large subunit binding proteins of *RUBISCO*, *NADPH* oxidoreductase and I and II-photosystem proteins were found with a significantly different expression during the HMs toxicity. Interestingly, these proteins are also regulatory to produce MT (Yu et al., 2018,b; Pardo-hernández et al., 2021).

## Conclusion and future perspective

Phytomelatonin has been found to be present in all plants, which has an important role in plants as a biostimulator that improves plant tolerance to both biotic and abiotic stress. The first layer of tolerance in HMs stress tolerance is the signal reception of HMs toxicity then transduction to other cells for HMs stress response. This fundamental communication in response to HMs stress is mediated by protein cipher, which plays a prime role in intracellular signal transduction, and activating signaling cascade. Proteomic analyses of various plants under HMs stress deciphered several molecular mechanisms underlying this cascade in the form of variation in protein level that enable or unable plant species for stress tolerance This work is of critical importance for developing stress-tolerant plants. However, a detailed study involving antioxidant mechanism, metals-regulated differential gene expression, and mineral transporters is needed to understand complex plant responses to metal toxicity.

## Author contributions

SA, MS, MK, BA, RG, and WZ designed the manuscript. SA, SAh, NZ, MR, and WZ wrote the manuscript. MN, RF, BA, RG, MS, and WZ revised the manuscript. All authors contributed to the article and approved the submitted version.

## Funding

This work was supported by the Science and Technology Department of Zhejiang Province (2022C02034), Collaborative Innovation Center for Modern Crop Production co-sponsored by Province and Ministry (CIC-MCP), and the Agriculture and Rural Affairs Department of Zhejiang Province (2022SNJF010).

## Conflict of interest

The authors declare that the research was conducted in the absence of any commercial or financial relationships that could be construed as a potential conflict of interest.

## Publisher’s note

All claims expressed in this article are solely those of the authors and do not necessarily represent those of their affiliated organizations, or those of the publisher, the editors and the reviewers. Any product that may be evaluated in this article, or claim that may be made by its manufacturer, is not guaranteed or endorsed by the publisher.
